# Ultra-Structure database design methodology for managing systems biology data and analyses

**DOI:** 10.1186/1471-2105-10-254

**Published:** 2009-08-19

**Authors:** Christopher W Maier, Jeffrey G Long, Bradley M Hemminger, Morgan C Giddings

**Affiliations:** 1 Department of Microbiology and Immunology, UNC Chapel Hill, NC, USA; 2 Independent Researcher, San Francisco, CA, USA; 3 School of Information and Library Science, UNC Chapel Hill, NC, USA; 4 Department of Biomedical Engineering, UNC Chapel Hill, NC, USA; 5 Department of Computer Science, UNC Chapel Hill, NC, USA

## Abstract

**Background:**

Modern, high-throughput biological experiments generate copious, heterogeneous, interconnected data sets. Research is dynamic, with frequently changing protocols, techniques, instruments, and file formats. Because of these factors, systems designed to manage and integrate modern biological data sets often end up as large, unwieldy databases that become difficult to maintain or evolve. The novel rule-based approach of the Ultra-Structure design methodology presents a potential solution to this problem. By representing both data and processes as formal rules within a database, an Ultra-Structure system constitutes a flexible framework that enables users to explicitly store domain knowledge in both a machine- and human-readable form. End users themselves can change the system's capabilities without programmer intervention, simply by altering database contents; no computer code or schemas need be modified. This provides flexibility in adapting to change, and allows integration of disparate, heterogenous data sets within a small core set of database tables, facilitating joint analysis and visualization without becoming unwieldy. Here, we examine the application of Ultra-Structure to our ongoing research program for the integration of large proteomic and genomic data sets (proteogenomic mapping).

**Results:**

We transitioned our proteogenomic mapping information system from a traditional entity-relationship design to one based on Ultra-Structure. Our system integrates tandem mass spectrum data, genomic annotation sets, and spectrum/peptide mappings, all within a small, general framework implemented within a standard relational database system. General software procedures driven by user-modifiable rules can perform tasks such as logical deduction and location-based computations. The system is not tied specifically to proteogenomic research, but is rather designed to accommodate virtually any kind of biological research.

**Conclusion:**

We find Ultra-Structure offers substantial benefits for biological information systems, the largest being the integration of diverse information sources into a common framework. This facilitates systems biology research by integrating data from disparate high-throughput techniques. It also enables us to readily incorporate new data types, sources, and domain knowledge with no change to the database structure or associated computer code. Ultra-Structure may be a significant step towards solving the hard problem of data management and integration in the systems biology era.

## Background

Biological research is an increasingly information-rich endeavor, with complex, heterogeneous data being generated at rates that outstrip the ability to readily manage, integrate, and analyze it. Experimental platforms like mass spectrometry (MS)-based proteomics can produce tens to hundreds of gigabytes of data in a single run comprising less than two days of machine time. A wide variety of similarly prodigious experimental approaches are in use by biologists. There are also a large and growing number of publicly accessible repositories for biological information, the largest being GenBank, UniProt, Ensembl, and the UCSC Genome Browser. Each of these resources may contribute critical knowledge or data toward solving a biological research problem, but integrating their diverse structures and data types into a unified analysis remains very difficult.

One of our research endeavors that has encountered these issues is proteogenomic analysis of the human genome. This uses proteomic data to examine the translation of RNAs to proteins on a genome-wide scale. We use computational techniques to map peptide-based MS data to their encoding genomic loci (GFS [1]). The approach is used to reveal alternatively spliced or frameshifted translation products that cannot be readily found by standard proteomic analysis methods. This project involves the melding of several data sources that are large and heterogenous: MS-based proteomic data, genome sequences, gene annotation sets, SNP sets, and more. Each MS data set may contain 10^5 ^or more individual spectra that must be analyzed in the context of one or more human genome sequences, each containing 10^9 ^nucleotides, along with multiple gene, EST, cDNA, and gene prediction annotation sets. Multiple spectral datasets are used, and they are available in a variety of formats depending on the source.

Not only are data sets heterogeneous and unwieldy, but the sources and types of data available often change as technology progresses. Add to these the frequent changes in file formats, analysis approaches, and pipelines, all of which contribute to the steep challenges to maintaining an information management platform. And change can go deeper than just new instruments or technologies employed; often, biological concepts change through time. For example, the concept of "gene" has undergone many changes since Mendel, and is currently experiencing another such change [2]. When biological concepts change, information systems built to those concepts must often undergo extensive modification, raising the specter of extensive software maintenance. For individual labs pursuing systems and genomic research, this is a daunting task.

When we set out to build an information system for the proteogenomic mapping project, these considerations led us to examine new means for managing, integrating, and analyzing project information. We discovered an intriguing approach developed by Jeff Long, called "Ultra-Structure", which employs a relational database system with a non-standard schema and code development approach to deal with issues of heterogeneity, complexity, and change [3,4]. This approach views all systems, regardless of their complexity, as the product of the "animation" of (sometimes large sets of) relatively simple rules (stored in a database), not unlike Wolfram's much more recent claims to "A New Kind of Science" [5]. However, unlike Wolfram's approach, Long's approach is oriented towards practical problem solving, and has for more than twenty years been applied to concrete challenges such as document analysis for nuclear technologies [6] and management of businesses [3,4]. Additionally, Long has also successfully experimented with its application to the representation of music and the analysis of games. When we encountered Ultra-Structure theory, it had not been applied to the biomedical and biological sciences, despite having properties well-suited to these fields, such as its ability to adapt to change and to integrate large heterogeneous datasets.

In this work, we examined whether Ultra-Structure could be practically applied to our proteogenomic annotation project, and then extended into other biological data management and integration tasks. Initially, we maintained two separate systems for this project, one using a traditional entity-relationship (ER) modeling approach, and the second built using Ultra-Structure principles. This allowed direct comparison of the approaches. While the Ultra-Structure system is not limited to just one use (in fact, we are already applying it to other uses), we focus here on illustrative examples drawn primarily from our proteogenomic annotation work.

The core of Ultra-Structure lies in expressing every piece of knowledge, information, or data as a "rule". Rules are managed in a standard relational database management system (RDBMS), but are organized in a unique way based on the concept of "ruleforms" (short for "rule format"). Each ruleform is a single table in the RDBMS, representing a single syntactical structure for the rules it contains. For example, a specific ruleform is used to declare the existence of entities of biological interest in a system (call them "BioEntities"), from small to large, including cells, molecules, DNA, membranes, and test tubes. All rules declaring BioEntities are then expressed with identical syntax within this ruleform. Another type of ruleform is then used to express relationships between BioEntities. This BioEntity Network ruleform uses a specific syntactical form to express binary relationships such as "Protein ABC is-a Tyrosine Kinase" or "Adenosine is-a Nucleoside". Each ruleform, while specific about the syntactical structure of the rules it represents, is very general with regard to the concepts those rules can be used to model. In both of the aforementioned ruleforms, there is nothing specific to any one field of biology encoded; they could be used equally well to express rules related to oncology research or proteogenomic annotation.

By representing everything about a system as rules (including data, processes, and ontologies), aspects of a system that are volatile are stored as data, rather than as database structure or computer code. The programming code implemented for a system, known as "animation procedures", is general, operating at a high level on the structure of the rules rather than their specific content. For the example of BioEntities given above, any computer code written to operate on a BioEntity operates on *any *kind of BioEntity, not just those specific to a particular research field.

The area of managing biological information and complexity is an active one. Several projects have investigated issues of database integration from the perspectives of data warehousing (GUS [7], Atlas [8], BioWarehouse [9]) and database view integration (K2/Kleisli [7,10], TAMBIS [11]). Some have utilized biological ontologies to manage biological information (TAMBIS [11], SEMEDA [12]). These warehousing approaches generally rely on the creation of a global schema, which is often large and complex, and dependent on the changing schemas of the databases they integrate. This can lead to negative consequences, as Stein related regarding the implosion of the Integrated Genome Database [13].

The "Semantic Web" is another approach to data integration that is gaining in popularity [14-16]. This approach relies on open standards (e.g., RDF, OWL) to present data and relationships in a consistent, machine-readable manner. Automated procedures can traverse data relationships across the Internet, allowing researchers (among others) to access and integrate related information from around the world [17]. Such semantic approaches are in a similar vein as our work, attempting to find novel ways to effectively deal with the vast amounts of biological data we are faced with. Ultra-Structure differs from the Semantic Web in that the latter is concerned with the machine-mediated exchange of data, whereas Ultra-Structure is focused on using a flexible rule-based system to manage local processes and data. Semantic web technologies could be overlaid on top of an Ultra-Structure system, allowing outside resources access to Ultra-Structure-managed data, thereby taking advantage of both approaches.

Nadkarni and colleagues actively develop systems using the alternative database design methodology known as EAV/CR (Entity-Attribute-Value with Classes and Relationships [18-20]), which has similarities to Ultra-Structure. Like Ultra-Structure, EAV/CR systems offer an insulation against change by storing volatile conceptual and structural information as data. However, the Ultra-Structure approach differs by also encoding information about behavior and processes as well, something these systems do not address. While our implementation stores gigabytes of project data (expressed as rules), it also implements processes such as the translation of an RNA transcript to a protein molecule, encoding the rules of translation (using several genetic codes) in the database. We have begun constructing a workflow management subsystem, tracking data analyses and processes using the same database structures. When domain knowledge about any of these processes changes, such as discovery of new alternative genetic codes, database rules are readily updated. Ultra-Structure is unique in this regard, as it can deal with information management on a variety of scales, including domain knowledge, experimental information, and biological and laboratory processes.

Here we examine in more depth the application of Ultra-Structure to proteogenomic mapping through the use of concrete implementation examples.

## Methods

Most approaches to information management model a system in terms of the concrete objects and relationships that are visible to the user or programmer. The perspective of Ultra-Structure is that these surface-level features are the results of ongoing, dynamic processes. These processes generate the objects, relationships, and attributes that we see as the outward manifestation of the system, and an Ultra-Structure system attempts to model these fundamental processes. Ultra-Structure comes from a background of process philosophy [21], which holds that processes, not objects, are the fundamental metaphysical constructs of the world.

In modern biological research, the surface-level features (kinds of objects, relationships between objects, etc.) are continually changing. For example, in a little over 30 years, DNA sequencing methods have progressed from early Maxam-Gilbert sequencing, through Sanger sequencing, to microarray sequencing, on up through the current state-of-the-art next-generation sequencing methods. When a database system is structured to directly mirror the data types produced by a changeable technology like this, it must be reconfigured each time the technology changes, along with sometimes extensive changes to the associated computer code. The result is that such databases will grow in complexity until they suffer from significant maintenance challenges, at which point it may be simpler to completely scrap the system and start over.

**Figure 1:**
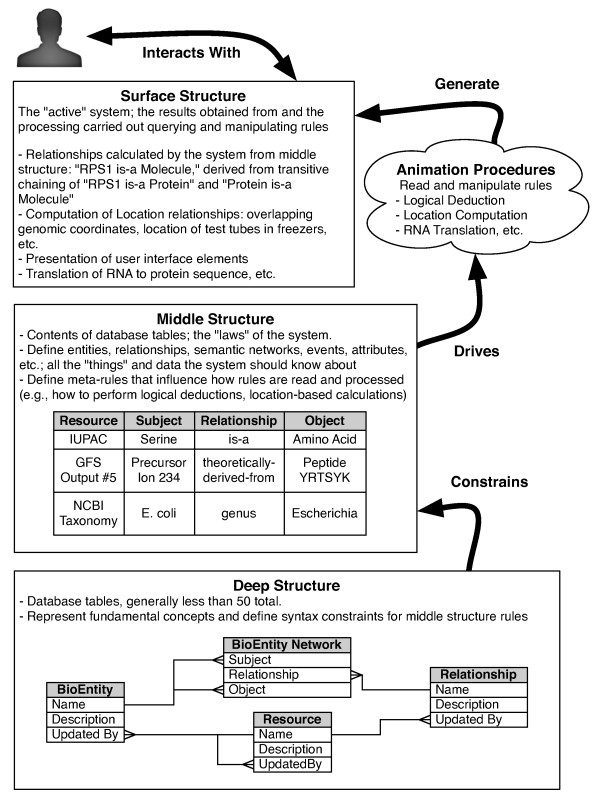
**Abstraction layers of the Ultra-Structure approach**. The "deep structure" (bottom box) abstracts the fundamental concepts of the system. It is implemented as tables in a standard relational database system. It serves to constrain the structure of rules that can be added to the system, known collectively as the "middle structure" (middle box). These rules, stored as rows in database tables, in turn drive the execution of generalized software code called "animation procedures", which act to generate the "surface structure", or the real world manifestation of the system (top box). It is this easily-perceived but constantly changing level of surface structure that is generally modeled in traditional ER design. The end user (shown at top) interacts with this aspect of the system.

To address this, Ultra-Structure introduces two primary levels of abstraction between the database management system and the "surface structure" that the user interacts with to store, analyze, and visualize their project data: the "deep structure" and the "middle structure" (Figure 1). (There are lower levels of abstraction beyond deep structure that may be of interest for readers interested in principles of notation [4,22].)

Below, we briefly cover the basics of the Ultra-Structure implementation. Rather than a full exposition, we cover further implementation details as part of the examples shown in the "Results" section.

### Rules

**Figure 2:**
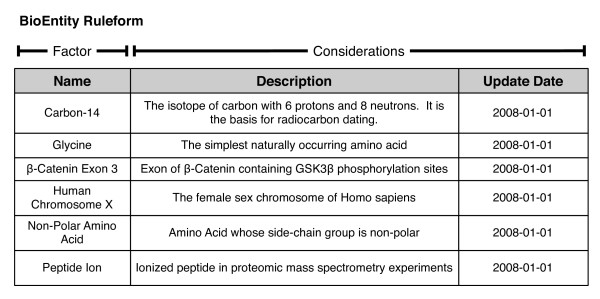
**Basic existential ruleform**. Ruleforms are tabular data structures that define the structure of rules. All columns of a ruleform can be divided into factors and considerations, which roughly correspond to the antecedent and consequent, respectively, of an "if/then"-style construction. Factors are the means by which rules are addressed and selected, and in practice are implemented as keys in a database system. The remainder of the columns are known as "considerations", and provide metadata about a rule, as well as additional information that may influence the ultimate processing of a rule. The ruleform shown here is a simplified version of the BioEntity ruleform, which is used to define the existence of concepts and entities that participate in various biological processes. It is also an example of an existential ruleform, which is used to declare the existence of entities in the system.

Rules are the core of Ultra-Structure, as they govern the fundamental ongoing processes that create the system being modeled. They are used to represent all changeable aspects of the system, including data, operational processes, attributes, etc. For example, simple declarative rules for objects in the system are shown in Figure 2.

In contrast to the "if/then" interpretation of rules in an expert system, Ultra-Structure rules are interpreted as "if/consider" constructs. When the conditions on the left-hand side of a rule – known as "factors" – are met, the system is instructed to consider the information of the right-hand side – "considerations" – in subsequent processing. This allows multiple rules to express considerations that apply to a given antecedent; as a result, we may conditionally modify how rules are interpreted.

For instance, in our system, one rule may state that in general, all sequence locations should be calculated relative to the 5' end of a chromosome. But, another rule may declare that short peptide sequences are declared relative to the start of the coding sequence for a gene. When rules are being interpreted, these two rules would present a conflict. However, since applicable rules are all considered before selecting an outcome, other rules in the system can influence processing, such as by defining an order of precedence so that the rules related to peptide locations take priority. While this has similarities to class inheritance in object-oriented systems, it is readily modifiable. If we later need to change the interpretation priority for rule types, only one rule need be modified that tells the system how to prioritize the considerations.

Rules are not completely free-form, though; they are subject to a variety of constraints, which are collectively known as the "deep structure".

### Deep Structure

The "deep structure" abstraction level is designed to represent those things about a domain that are fixed and unchanging. Technically, the deep structure consists of the syntactical and semantic forms for expressing rules relevant to the application domain (the ruleforms), as well as the associated computer code (animation procedures) that manipulates those rules. Ruleforms specify all the logically possible forms that a rule may take: "Ruleforms are to collections of rules as numbers are to collections of things. ... Ruleforms abstract morphology, while numbers abstract quantity; in a sense, ruleforms model the geometry of rules." [3] In practice, ruleforms are implemented as database tables: the ruleform itself is the table definition, and individual rules that conform to that ruleform are rows within that table. Factors are implemented as a primary key (or unique) constraint on the ruleform table; considerations (including metadata, such as "update date") are then the remainder of the columns of the table. Standard database system query mechanisms can be leveraged to quickly find applicable factors (keys) for given system inputs, and furthermore, modern database systems can easily handle and query millions of rules. The ruleform abstraction allows their use for many distinct functions, while keeping the number of tables low and comprehensible.

The development of ruleforms for a given domain comes about from following an iterative development process, starting with a high-level process-oriented perspective, implementation, and refinement. Across broad domains for which Ultra-Structure systems have been implemented, typical systems utilize 50 or fewer ruleforms (and hence database tables).

While ruleforms can prescribe the forms of all rules of a system, the rules themselves are inanimate; they need to be interpreted in order to produce the desired behavior of the system. The primary feature of the associated animation procedures is that they are implemented in a general way without detailed domain knowledge, allowing the system to accommodate new information or procedures without changing the underlying software. Since an Ultra-Structure system encodes information as formal rules in a database, including how rules are to be interpreted, this simplifies the needs for animation procedures, so that they must only deal with control logic, such as which ruleforms to inspect and when, and how to get data into and out of the system. Specific actions carried out by the software are driven by the contents of the rules being operated on; changing the rules changes the actions performed by the system. As a result, animation procedure code will not need to be changed as domain knowledge changes. In this regard, the animation procedures of Ultra-Structure are similar to the inference engines found in expert systems, but differ in the fact that they access different tables for different kinds of rules.

For example, one common need of many biological research systems is to calculate spatial or structural relationships between two items, such as whether a nucleotide is within a gene (and what gene that is), or how close two amino acid residues are along a protein string. In our system, an animation procedure deals with any type of arbitrary spatial calculation on linear strings, regardless of what type they are. The biology-specific calculations, such as calculating all exons within a gene, are determined by protocol rules in the system.

Together, the collection of ruleforms and the animation procedures that operate on them constitute the deep structure of an Ultra-Structure system. In the case of our system, we have implemented (and continue to refine) a deep structure which aims to capture the fundamental computational realities associated with biological research.

An example of deep structure in our system arises from the need to declare the existence of entities such as genes, proteins, cells, samples, and amino acids. The BioEntity ruleform from Figure 2 is an example of an "existential ruleform", which is used to declare the existence of some concept or entity in the system: the "things" the system knows about. Our BioEntity ruleform defines things that participate in biological processes, or are otherwise of biological interest. This includes a broad diversity of concepts, from the isotope ^14^C and the third exon of the *β*-catenin gene, to broad categories, such as "Non-Polar Amino Acids". A group of BioEntities is itself considered a proper BioEntity because it is subject to the same kinds of rules; rules that apply to the group also apply to the members of that group.

This ruleform defines the structure for rules declaring the existence of biological entities of any kind. This is in contrast to a more standard RDBMS, where different types of biological entities would typically be stored in separate tables. Here, if a new type of biological entity comes into play later, there is no change to the underling system; the "BioEntity" ruleform can accommodate nearly any type of thing we might care to declare and track in the system.

While the "things" in a pure entity-attribute-value system might all be formally represented as generic "Entities" or "Objects", this is not the case in an Ultra-Structure system; everything in our universe of discourse is not a BioEntity. Other existential ruleforms for our system are shown in Table 1, and include foundational concepts such as Resources, Attributes, Locations, and Units of measurement.

**Table 1:** Existential Ruleforms

**Ruleform**	**Description**	**Examples**
BioEntity	Things that participate in biological processes or are otherwise of biological interest	amino acids, chromosomes, gene annotations

Resource	A source of information in the system	people, lab groups, software, books, output files of computational analyses

Event	Instances of some process or occurrence	experiments, software invocations, biological processes

Attribute	Facets of entities that are of in terest	mass, length, atomic number, statistical scores, GenBank accession number

Relationship	Binary relationships (predicates) that can exist between entities of the system	is-a, part-of, has-exon, overlaps

Location	General idea of a location or address; where an entity can be found in a variety of spaces	genomic locations, lab freezer locations, intracellular locations, geographic coordinates

Location Context	A location space; the framework in which a Location can sensibly be interpreted	18th draft human genome coordinate, 17th draft human genome coordinate, Giddings Lab freezer, Earth latitude and longitude

Unit	A unit of measurement	dalton, kilogram, meter

Several existential ruleforms (declaring the existence of various entities in the system) are described, with illustrative examples.

**Figure 3:**
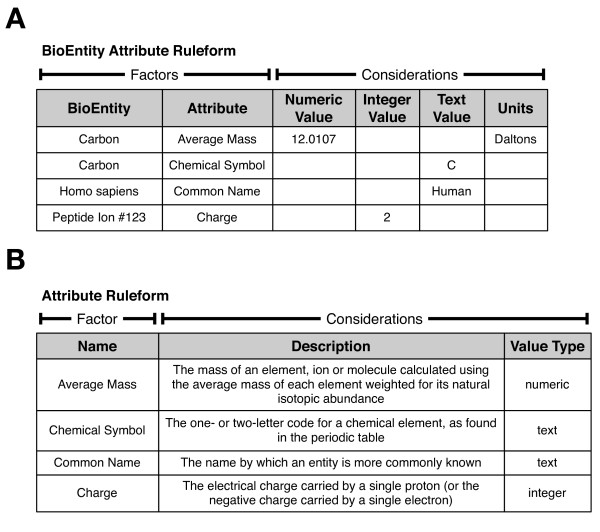
**Attributes**. Our Ultra-Structure system represents the various attributes of entities using ruleforms (tables) similar to these. Panel A: Simplified BioEntity Attribute ruleform. The factors include a BioEntity and an Attribute, while the considerations include columns in which typed values are stored. These rules can be read in plain English: "the average mass of carbon is 12.0107 daltons", and so on. Panel B: Attribute ruleform. This existential ruleform is used to define various Attributes, similar to how the BioEntity ruleform defines BioEntities. The "Value Type" consideration is used to allow software procedures to find the appropriate value column for a particular Attribute. For instance, the fact that the "Average Mass" Attribute has a value type of "numeric" indicates that an animation procedure would look in the "Numeric Value" column of the BioEntity Attribute ruleform to find the average mass of Carbon.

In addition to the declared objects like BioEntities, there can be a set of associated ruleforms that express attributes for them. For example, in proteomic research, the masses of molecules must be tracked. These can be expressed in different ways, based on how isotope distributions are accounted for, including "monoisotopic mass", "average isotopic mass", or "most abundant isotopic mass". We express this information using the BioEntity Attribute ruleform shown in Figure 3a, which is based on Entity-Attribute-Value design (EAV; also known as Object-Attribute-Value, or OAV). The factors of this ruleform include a BioEntity and an Attribute, the latter defined with an existential ruleform shown in Figure 3b. Attributes can track any kind of arbitrary information about an entity. There are multiple consideration columns present to effectively represent values of different data types, but in this case, constraints ensure that only one may be non-null, reflected by the "value type" consideration of the Attribute ruleform. In addition to unconstrained columns containing numeric or text values (as shown in Figure 3a), other columns may refer to other existential ruleforms, allowing different types of existential entities to be related to one another. Each type of existential ruleform may have an associated Attribute ruleform (e.g., Resource Attribute, Event Attribute).

**Figure 4:**
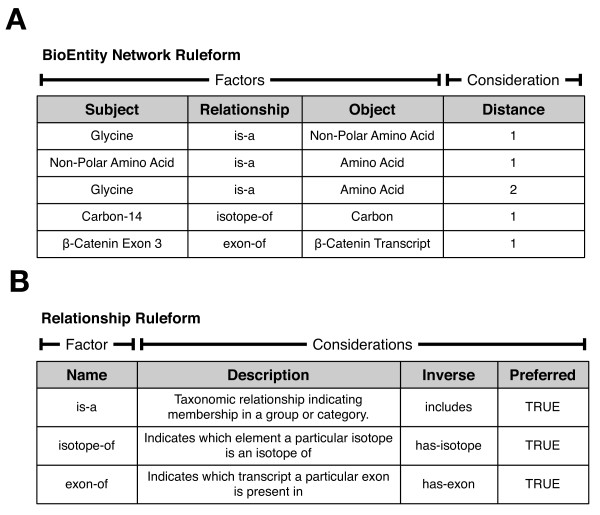
**Basic BioEntity Network and Relationship ruleforms**. Panel A: The BioEntity Network ruleform (simplified for clarity) exemplifies network ruleforms by defining relationships between BioEntities. Here, "Subject" and "Object" both refer to BioEntities, while "Relationship" refers to rules defined in the "Relationship" ruleform (Panel B). Each rule specifies a single binary relationship. The "Distance" consideration reflects how far away from each other in the network the two BioEntities are. In the rules shown, "Glycine" is two steps away from "Amino Acid", going first through "Non-Polar Amino Acid". Rules of distance greater than 1 are generated automatically via logical deduction. Panel B: The Relationship ruleform specifies all valid binary relationships that can exist between entities in the system. The "Inverse" consideration indicates the Relationship to use in the reverse formulation of a rule; for example, if "Carbon is-a Element", then "Element includes Carbon". The "Preferred" consideration is a Boolean flag that indicates Relationships that are to be used when entering original (as opposed to automatically deduced) rules.

Once an entity is declared to exist in an existential ruleform, it can be combined with other entities to create new "network" rules that express relationships between them. Figure 4a shows a simplified example of a network ruleform in our system. It expresses arbitrary binary relationships between BioEntities using "subject/predicate/object"-style triples, where "subject" and "object" can be any entry represented in the BioEntity ruleform; the predicate can be any relationship defined in the Relationship existential ruleform (shown in Figure 4b). This network ruleform can be used to specify which entities are the members of a group, the group itself being a BioEntity. In this framework, it is possible to build up networks, hierarchies, and relationships between a variety of entities easily. Users can directly add new Relationship rules to the system to define whatever associations they might need, without modifying the database structure itself.

Though omitted in the figures for clarity, several ruleforms have an additional factor called "Resource", used for system provenance. The BioEntity Attribute ruleform has a Resource factor, which allows recording who declared the average mass of carbon to be 12.0107 daltons, or which MS analysis file contained the information that Peptide Ion 123 has a charge value of 2. Since the Resource is a factor (and a rule's combined factors must be unique in that ruleform), this allows the storage of potentially conflicting information in separate rules. This addresses the fact that information in scientific endeavors is often tentative and/or integrated from a variety of sources. Otherwise, information stored in the system would be limited to dogmatic declarations of concrete fact. The Resource factor is used wherever the declaration of data or rules may be tentative, conflicting, or changing.

Other ruleforms in our system are used to define additional kinds of rules: "authorization rules" indicate what combinations of information are valid or permitted; "meta-rules" describe how to read other rules; and "protocol rules" specify sequences of actions and can be used to implement complex processes and workflows.

### Middle Structure

The next layer of abstraction, the "middle structure", can best be thought of as representing the "laws" of the system; that is, the specific rules and constraints that govern a system's operation. The middle structure is the content of the deep structure's ruleforms. This represents the types of relationships that may occur between data in the system, and the processes that data undergo during analysis or query. In our system, the middle structure declares the types of data kept about our biological research operations, the relationships between them, and the kinds of operations that can occur on the data. Whenever procedures, file formats, or data types change, the middle structure is changed to reflect this. Often the relevant database system changes can be made by the non-programmer, by modifying the database entries that comprise the rules of the system. This puts the task of regular maintenance more fully into the hands of the systems users, who are the subject experts, rather than (usually) third-party programmers.

In our system the middle structure encompasses BioEntity rules defining various genome annotation sets, mass spectra, and amino acids; Resource rules defining our GFS software, genome annotation providers, and individual GFS analysis output files; Location rules defining specific regions of genomic sequence for annotations as well as GFS-mapped peptide locations; and several others. Another lab performing bacterial community analyses – a wholly different research area – could take the same deep structure, but instead populate the middle structure with a separate set of rules, such as BioEntity rules defining restriction enzymes and bacterial species, BioEntity Network rules reflecting taxonomic relationships between bacterial species, Events denoting specific analysis experiments, and Locations representing lab freezer locations of samples. We are in fact implementing a system for bacterial community analysis with collaborators, using our proteogenomic deep structure as a basis.

One brief example is in order. We regularly exploit the concept of "overlap" between two sequences against a particular genome location, such as to find where peptides overlap annotated exons or genes. The procedures for calculating overlap are not part of the structure or code of the system, since it reflects a specific use case; instead, it is represented by a small number of rules that are readily changed or added to as demands warrant. Many other Relationships besides simple "overlap" may be of interest, such as "located downstream" or "contains"; these and others can be defined by the end user. Ultimately, by not over-specifying the deep structure of the system, users are able to modify the middle structure to obtain the surface structure they desire.

### Surface Structure

In an Ultra-Structure system, the "surface structure" – the actual appearance of the real-world system – is not explicitly modeled or stored anywhere; rather, animation procedures driven by the content of the rule base (the middle structure) generate it. Surface structure is volatile in biological research, where experimental protocols change, new data types become available, and concepts are continually revised in light of new information. As a result, information systems modeled on surface structure directly are themselves volatile, lending to maintenance issues.

An example of surface structure generated by our system can be seen in user interfaces derived from the combination of middle structure rules with general animation procedures. For example, when a user queries the system to find a list of existing genomic annotations that positionally coincide with a peptide match from a tandem MS (MS/MS) experiment, the Ultra-Structure system dynamically generates the interface and resulting lists by examining its rules to determine what it means for genomic regions to overlap, as well as what values should be queried for and then returned to the user.

In our system, the surface structure is generated using animation procedures that interact with the RDBMS middle and deep structure. Our system is built on the open source PostgreSQL database server [23]. Animation procedures are implemented as internal database procedures written in either PL/pgSQL (a PostgreSQL-specific procedural language) or SQL (though any of the several procedural languages available for PostgreSQL could be used), or as client-side methods written in Java, using the Hibernate [24] object-relational mapping (ORM) library, or a combination of both. In general, animation procedures that perform large amounts of database processing, or that require a great deal of dynamic query generation are implemented as internal database procedures, while simpler procedures, and those whose execution requires control or data structures that are difficult to implement in the database are performed on the client side. We are also developing a web interface to the system using Stripes [25], an open source web application framework for Java. Examples of some prototype interfaces can be seen online at . Some example SQL queries are provided in Additional file 1 for interested readers. Prototype code for the system is also provided in Additional file 2.

## Results

At the beginning of this project, we developed the Ultra-Structure system while also maintaining a traditional ER-modeled database for the project. The latter was in place until it became clear that the Ultra-Structure system would reach sufficient utility for day-to-day use. The ER system developed to contain 66 individual tables representing everything from MS datasets to analysis results to various genomic annotation datasets downloaded from UCSC's genome resources [26]. The ER approach provided a quick start-up due to our familiarity with it at the time, but as the scope of the project evolved, shortcomings became apparent. For instance, its structure assumed all annotations and proteomic data had coordinates relative to a single genome. The subsequent availability of multiple genome drafts along with genomes of multiple individuals [27,28], necessitated substantial redesign, which would have resulted in more tables to track and coordinate all the different versions of genomes against annotations and proteomic runs. Other shortcomings were more fundamental; for example, it was necessary to build into the ER model some fixed representation for a "gene". However, the definition of a gene is changing as more is uncovered about processes such as trans-splicing [29], where one messenger RNA is produced by concatenating exons from separate pre-mRNAs.

On the other hand, implementation of the Ultra-Structure system was at first challenging, because the design process required a new way of thinking that focused on separating universal features of the system (such as the need to declare the existence of biological entities), from the changeable features (such as the need to query for exons overlapping a given peptide sequence). The process has been iterative; we started with a core of ruleforms (tables) representing the basics such as BioEntities, networking of BioEntities, Resources, and Events. As we began implementing rules and features, we came upon the need for new ruleforms, such as one representing Location information, and another representing BioEntity Attributes. As we have become more adept at the Ultra-Structure approach, each refinement to the system has occurred more quickly, and resulted in a more coherent design.

The resulting Ultra-Structure system stores all the data that our original database could, and stores it in a more comprehensible manner, using fewer tables (approximately 40). We have translated procedures from large, complex software functions into Ultra-Structure animation procedures that are smaller, easier to comprehend, and driven by rules in the database that can be altered to extend the capabilities of the system. Using our proteogenomic annotation project as an example, we describe the modeling processes and considerations that we faced in transitioning from a traditional ER-modeled system to one based on Ultra-Structure. Rather than trying to be comprehensive in the description of the system, we focus in on several examples that illustrate the types of changes and considerations made. Further detail can be gleaned by examination of the system and documentation itself, prototype code for which is attached as a Supplemental File.

### Example 1: Modeling and implementing hierarchies of mass spectrometry data

**Figure 5:**
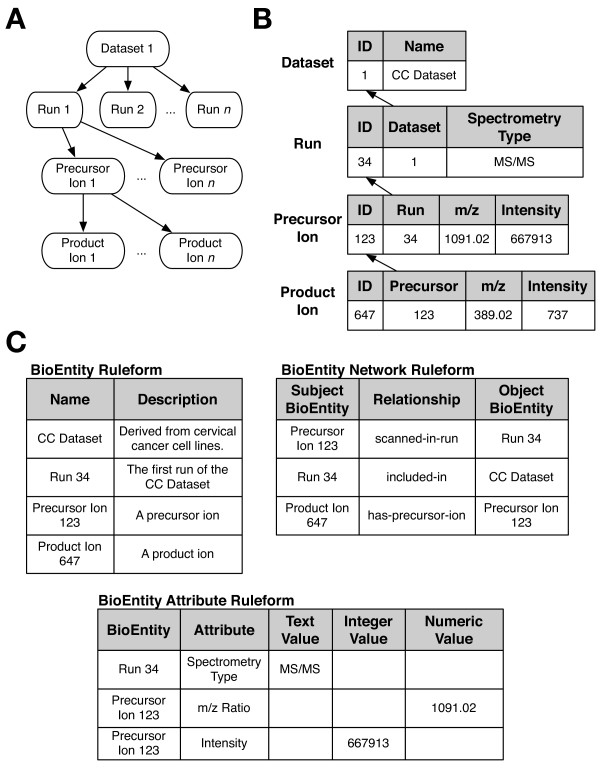
**Representing hierarchical information in Ultra-Structure**. Panel A: conceptual representation of hierarchical information. Briefly, a Dataset consists of multiple Runs, each of which has a number of Precursor Ions, which in turn can produce multiple Product Ions. Panel B: one possible schema for this hierarchy using traditional ER modeling. Each level of the hierarchy is represented by a separate table. Various attributes of entities are represented using a number of additional columns. The structure of the hierarchy (i.e., the inter-level links) are represented using foreign key relationships, shown here with arrows extending from the referring data to their referents. For instance, each Run is from a single Dataset (indicated by its "Dataset" column), while a single Dataset may have several Runs that refer to it. Panel C: reformulation of the same information using Ultra-Structure ruleforms. Entities at all levels of the hierarchy are declared in the BioEntity existential ruleform. The various attributes of each entity are represented by individual BioEntity Attribute rules. Finally, the structure of the hierarchy is declared using BioEntity Network rules. The specific nature of the links between entities is declared explicitly, using the appropriate Relationship. These same tables could be used to represent any other hierarchy of BioEntities, regardless of depth, whereas the traditional ER model would require as many tables as there are hierarchical levels. As mentioned earlier, the actual BioEntity Attribute and BioEntity Network ruleforms have an additional Resource factor (which in this case would be used to denote the particular data file the data come from) as well as additional considerations that are omitted here for simplicity and clarity.

Hierarchies are ubiquitous in biological research, both in the subject of the research (proteins are made up of subunits, which are made up of amino acids, which are made up of functional groups, which are made up of atoms) and in the artifacts of the research itself (experiments are broken down into subtasks, which are composed of individual steps). These can be represented with ER modeling methods by creating a series of tables linked by integrity constraints, with each table representing entities at successively deeper levels of the hierarchy. Mass spectrometry data is naturally hierarchical, with an experiment usually comprised of a group of separate LC-MS (liquid chromatography-mass spectrometry) runs, each of which consists of multiple spectra, which in turn are each comprised of series of peaks (Figure 5a). Such data can be represented in a standard ER design as shown in Figure 5b, where the hierarchy of the data is mirrored by a hierarchy of tables in the database. This has the benefit of being an intuitive and relatively natural representation.

Ultra-Structure instead represents this hierarchy by abstracting the concept of networks of BioEntities in a general manner. A grouping of data points such as a spectrum is a BioEntity, as are each of the data points themselves (Figure 5c, upper left). The network itself is implemented in a new ruleform (table), the BioEntity Network (Figure 5c, upper right). This ruleform captures a high level, general aspect of the system: that there exist relationships between entities that participate in biological processes or interactions. For the example of MS data, we define a relationship using the Relationship ruleform (Figure 4b) such as "has-precursor-ion", then use this to relate each specific product ion with the specific precursor ion it was derived from. In a similar way, we can use an "included-in" Relationship to associate a particular run with the dataset it is a part of.

These relationships existed in the ER-based system as foreign key constraints. However, a foreign key can only indicate that data from one table has an association with data from another table; it cannot formally and explicitly specify the nature of that relationship (e.g., that it represents a "has-precursor-ion" relationship). In Ultra-Structure, the relationship is encoded as data (i.e., as a rule) within the middle structure of the system, providing explicit information about the nature of the relationship to human users and software. New relationships are created by adding new rules to the Relationship ruleform, which are immediately available for creating new rules in the BioEntity Network ruleform. Nearly any arbitrary network of BioEntities can be formed, without changing the database structure. It is straightforward to group MS data in new ways, for example to accommodate gel-based data instead of LC-MS runs. In contrast, for the ER model, new representations or hierarchy models are likely to involve changing the database schema itself, which in turn impacts the computer code that interacts with it.

Each BioEntity (ions, runs, datasets, etc.) may have one or more attributes. For example, ion peaks may have an intensity and an associated mass, as well as an electrical charge. We represent such attributes using a separate ruleform, "BioEntity Attribute" (Figure 5c, bottom). Users can make the system aware of new Attributes by adding the appropriate rules in the Attributes ruleform (Figure 3b). As such, the BioEntity Attribute ruleform can be used to express the value of any Attribute of any BioEntity. This attribute representation is similar to the EAV/CR approach [18], but in the latter, the attributes of all kinds of objects (regardless of their class) are represented using an identical table structure, whereas Ultra-Structure attribute tables are tailored to each semantically distinct type of entity (BioEntity, Resource, Event, etc.), reflecting fundamental differences in their use in the system. For example, the BioEntity Attribute ruleform has a Resource factor that facilitates provenance, which may not be necessary for Attributes on other kinds of objects.

Since Ultra-Structure ruleforms represent a higher-level abstraction than ER-modeled tables, these three tables can now be used to represent much more than just MS/MS datasets. Indeed, the only things the original ER tables could represent were MS/MS datasets; to begin using another type of dataset would have required remodeling the existing tables or adding new tables. This flexibility of the Ultra-Structure approach will be demonstrated next.

### Example 2: Representing genomic features

One aspect of our project is to map the MS-analyzed peptide spectra to location(s) on one or more human genome sequences, then correlate those locations with features such as genes and exons that are part of publicly available annotation sets.

**Figure 6:**
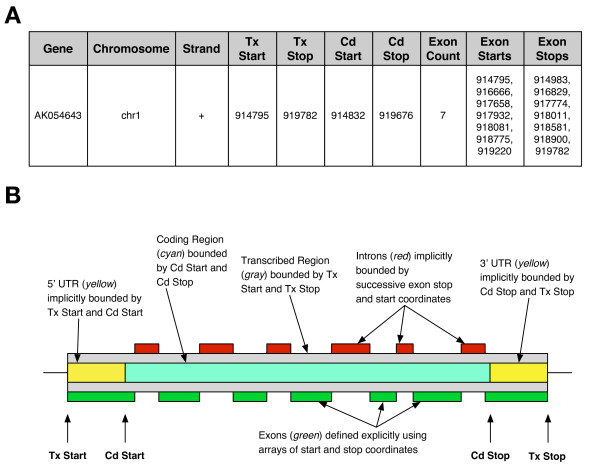
**Different representations of genomic regions**. Panel A shows an entry from the Known Genes dataset from UCSC, using its native database structure. Here, "Tx" stands for "Translation" and "Cd" for "Coding"; thus "Tx Start" and "Tx Stop" define the bounds for the particular transcript's transcribed region, just as "Cd Start" and "Cd Stop" define a coding region. Panel B illustrates how various annotations of interest are represented using this data structure (the figure does not represent any particular gene, and is not to scale). The transcribed region and coding region are both explicitly defined, while untranslated regions must be inferred. Exons are represented altogether differently, using arrays of start and stop positions. Introns must be inferred as regions lying between these exons. All these types of annotations are of interest to researchers, but the variety of representations used here pose challenges to querying. The data structure also assumes that all genes will fit this basic structure, which may not always be the case (e.g., trans-spliced genes can be composed of segments from different chromosomes).

Our ER database implemented a problem-specific table structure, mirroring the MySQL database dumps from UCSC [26], as shown in Figure 6a. We found this design to have two limitations, illustrated in Figure 6b. First, the table structure makes a specific assumption about the types of information that must be recorded for a gene, and how these are related to one another. If new features or structures for a gene are uncovered, such as a new splicing regulator in an intron, then the table must be redesigned, along with all the computer code written to interact with it. Second, some features of a gene, such as introns, are not made explicit in this model, but must be derived by calculations using other features. This makes it harder for a human to review the source data in the database, and makes errors in the data or the code that interprets the data more likely.

The Ultra-Structure approach instead encourages taking a step back and looking more generally at the problem of representing annotation data. We implemented a solution that took advantage of the existing ruleforms, considerably streamlining the representation. At its core, the approach declares that all annotations are simply BioEntities, such as "UCSC Known Gene Transcript Alignment uc001fet.1 Exon 1" or "Ensembl Transcript ENST00000002125 5' UTR". As such, the abstract entities that the annotations represent (a particular exon, or a particular untranslated region) are declared independently of specific coordinates in an arbitrary genome draft. This facilitates both representational efficiency and biological interpretation, because some annotations can appear in different drafts of a genome, and across genomes (e.g., mouse and human). In essence, this abstraction provides an independent existence for the concepts associated with particular genomic features. Researchers or computer code can thus interact broadly and in a relational manner with them, without having to reference specific genomic coordinates, just as it is possible to talk about a building on a university campus by name and relate it to nearby buildings without having to refer to street addresses or geographic coordinates.

Once established as BioEntities, annotations can then be related to one another using BioEntity Network rules, expressing that this exon is part of that transcript, for example. We need only declare the types of relationships that describe these connections. Using this representation, we can easily maintain abstract groupings or hierarchies of annotations that are biologically relevant. For example, we use this to create a grouping for all annotations belonging to the set of "Known Gene Annotations for Human Genome Draft 18". We can also use it to create groups such as "Human Genome Draft 18 Exons" or "Transcripts Associated with Breast Cancer". Representing all these open-ended grouping choices using a traditional ER model requires additional tables and associated infrastructure, while Ultra-Structure provides for this capability with no further alterations of the database's schema.

#### Example 3: Representing genomic locations and positions

Our project also needs to express discrete genomic coordinates for annotations and perform computations such as determining which (if any) genes a particular peptide occurs in. Our original ER system represented genomic locations similarly to the UCSC tables, capturing the surface structure of the problem in the database schema (e.g., a "genomic region" table with columns for specific attributes such as "chromosome", "strand", "start", and "stop").

**Figure 7:**
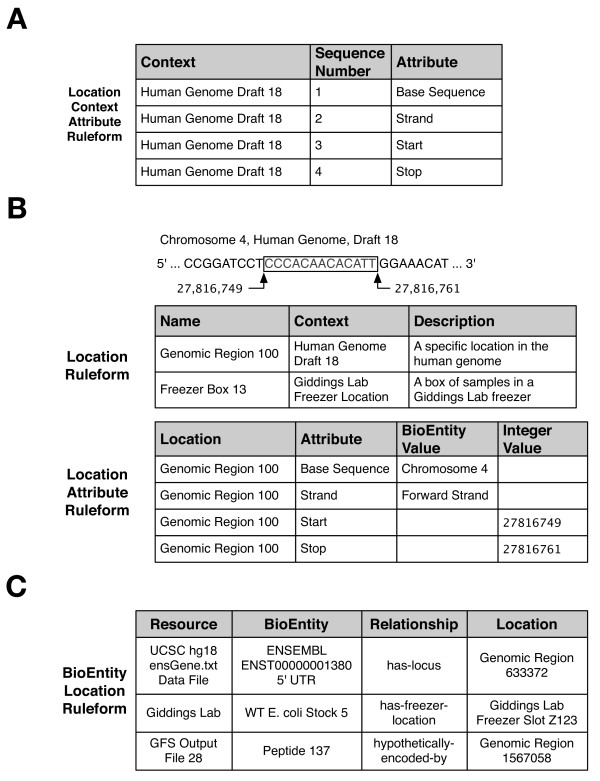
**Location-related ruleforms**. The Location Context Attribute ruleform is used to specify what kinds of Attributes a Location in the given context is allowed to have; these can be thought of as defining the internal structure of the Location. In Panel A, four rules specify that Locations in the 18th draft of the human genome must have four coordinates. "Base Sequence" here will refer to a chromosome, but can be used in other Location Contexts to stand for some sequence a Location may be defined on, such as a cloning vector or protein sequence. "Strand" will refer to either the forward or reverse DNA strand, while "Start" and "Stop" refer to the numerical coordinates where the Location will begin and end. This can be seen in Panel B, where the boxed region of the genome shown is represented as rules in the Location and Location Attribute ruleforms. First, a new Location must be defined, named "Genomic Region 100" (it may be anything, so long as it is unique among all Locations). This Location can now have values defined for each coordinate specified in the Location Context Attribute ruleform for an 18th draft genomic region by using Location Attribute rules, as shown. Finally, Panel C illustrates the BioEntity Location ruleform, which is used to define the kinds of Relationships that BioEntities can have with various Locations. In this sense, it is similar to the BioEntity Network ruleform, with the difference that here we relate a BioEntity and Location, not two BioEntities. This ruleform is another example of a Resource-qualified ruleform; it is important to know who says a particular annotation is located at a specific genomic location, just as it is important to allow for the fact that different proteogenomic analysis methods may map the same peptide to several places on the genome.

In designing the Ultra-Structure representation for locations, our goal was an abstract and general representation of locational information, flexible enough to represent other types of location information besides genomic regions. The result is a Location existential ruleform and several accessory ruleforms (Figure 7) that can represent many types of locations common to biological research: subcellular organelles, motifs and regions in peptide strands, test tubes in lab freezers, internet addresses of datasets, and even word and concept locations within journal articles and other text mining inputs.

The ruleform uses a consideration called "Context", which is a reference to the Location Context ruleform, specifying the frame of reference in which a specific Location is valid. For instance, a genomic coordinate for the 18th draft of the human genome can only be interpreted in the context of the 18th draft; it makes no sense in the context of the 17th draft, since the underlying genome sequence is different. Similarly, the location of a test tube in a lab freezer will not make sense for calculations on genomic coordinates. The Location Context Attribute is then used to define the coordinate space of Locations for a particular context. For example, this allows specification that a "Genomic Coordinate" context must define a "chromosome", "strand", "start position", and "stop position", or that a "Freezer Location" context for protein samples is defined in terms of "room", "freezer", "shelf", "rack", "box", and "slot". The Location Attribute ruleform is then used to store a specific Location's coordinates, using a form similar to the BioEntity Attribute ruleform. For example, "Genomic Location 100" is described by four rule entries: one for chromosome (or "Base Sequence"), one for directionality (or "Strand"), and one each for "Start" and "Stop" positions, as seen in Figure 7b.

Specific Locations, such as "Genomic Location 100", maintain a separate existence from any BioEntity that may refer to them. Just as an annotation (or any other kind of entity) can have multiple Locations (e.g., duplications, different drafts, different genomes), so too can a single Location correspond to several annotations.

We then use the BioEntity Location ruleform to connect gene annotation BioEntities to their associated Locations (Figure 7c). But it is not limited to that use; it is also used to associate matched peptides from mass spectrometric analyses to their genomic location. By implementing a solution for one location-related purpose, we get a solution to another location-based problem essentially "for free".

### Example 4: Animation procedures for generalized location calculations

As mentioned before, animation procedures perform procedural tasks without being programmed using any case or problem-specific knowledge (except as may be appropriate for that broad family of systems). One example is mapping MS/MS peptide data to genome coordinates, and then finding out what existing annotations say about those coordinates. For this task, we need to be able to calculate Relationships between Locations, such as "contains", "before", or "after". Some MS/MS data may map a peptide with high confidence to a particular nucleotide sequence on chromosome 17; we will want to find all existing genomic annotations that contain, are contained by, overlap the 3' end of, or are located downstream of this location (to name four possibilities). There can be many other relationships that are interesting, and it is difficult to predict ahead of time all the types of relationships users may want to examine. But in the end, we are always dealing with arbitrary calculations on two Locations that share a Relationship, in order to find any relevant associated BioEntities.

**Figure 8:**
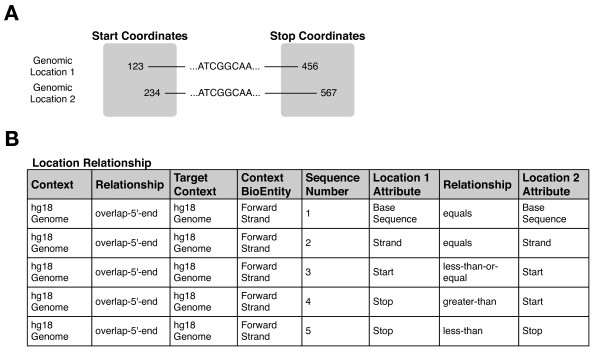
**Example ruleform for querying Locations by Relationship**. Panel A: schematic of two locations from the 18th draft of the human genome declared in the system to have some overlap, with Genomic Location 1 overlapping the 5' end of Genomic Location 2. This relationship can be specified in terms of relationships between corresponding pairs of Attributes of those Locations. Panel B: We make the types of relationships we wish to query explicit, and can now allow us to perform queries based on Locations. For example, we define here a set of 5 rules that specify a relationship of "Location 1 overlaps the 5'-end of Location 2" that holds between Locations in the 18th draft of the human genome (specified by the "Context" and "Target Context" columns). Other ruleforms (not shown) contain meta-rules that ensure the appropriate rules are chosen for a given scenario. In this case, the rules for determining a 5' overlap depend on what nucleic acid strand (forward or reverse) a given genomic region lies; here we show the rules that apply for the forward strand only. These rules specify what relationships must hold between attributes of our query Location ("Location 1 Attribute" consideration) and those of any other Location ("Location 2 Attribute" consideration) whose 5' region is overlapped by our query Location. For instance, the first rule states that both Locations must be on the same sequence (i.e., chromosome), while the second states that they must be on the same strand. The remaining rules constrain relationships between the start and stop positions of the Locations such that the desired relationship holds. Any kind of relationship that can be expressed in terms of relationships between Location Attributes can be represented in this way.

Using the Location-related ruleforms as the basis, we implemented a general animation procedure for performing coordinate-based Location calculations. For example, we know that genomic region A contains genomic region B if these regions are on the same strand of the same chromosome, and the start and stop positions of B are between those of A. This is defined explicitly as a set of rules in the Location Relationship ruleform that define a containment Relationship (Figure 8b). If the rules for containment change (e.g., if coordinate systems for storing genomes are modified), the only changes that need be made are to the rules that define the Relationship. Other Relationships can be similarly defined, such as "overlaps 3' end", "is downstream of", and "is upstream of".

**Figure 9:**
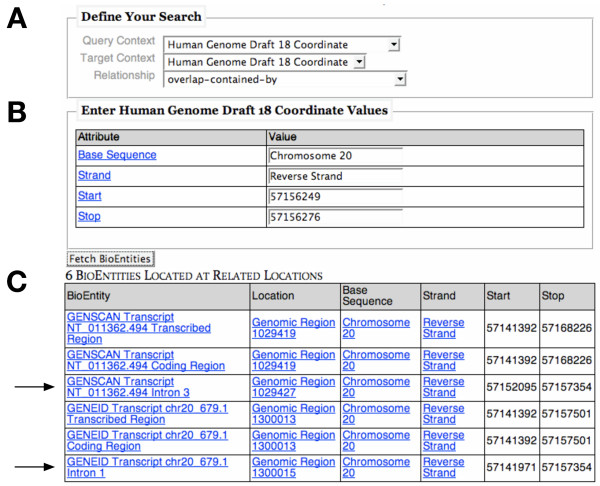
**Location-finding interface**. This interface for querying Locations in our Ultra-Structure system is generated based on the contents of the rulebase and user input. Here, the user has a region of the genome that has been mapped as the source of a peptide detected by MS/MS analysis and she wishes to see what other annotations contain this mapped region. Initially, only panel A is shown. The region the user has is the "query location"; in the top selection box, the user indicates that it comes from the 18th draft of the human genome. The user indicates she is interested in other annotations from the same draft with the drop-down box labeled "Target Context". Finally, based on the relationships the system knows that can exist between the given Query Context and Target Context (defined by rules), the user selects a Relationship; here, she chooses "overlap-contained-by" to indicate that she wants all Locations that fully contain her query location. Upon selecting the Query Context, the interface in Panel B appears, requesting details pertinent to a Location of the chosen Context. Since the user has a genomic location, the system asks for details such as "Base Sequence" (e.g., a chromosome), and "Strand". When the user enters this information and presses the "Fetch BioEntities" button, the system reads the rules that govern the "overlap-contained-by" Relationship between Locations from the 18th draft of the human genome, finds all the Locations that satisfy that Relationship, and returns a list of all BioEntities that are associated with those Locations. In this scenario, six such BioEntities are returned, along with detailed Location information. The two rows indicated by the arrows represent annotated introns (suggesting heretofore unknown translational activity), while the other four represent annotations that themselves contain the introns (and, by extension, the user's query Location).

The animation procedure takes as input a Location, a Location Context (indicating the kind of Locations to search for), and a Relationship. The Location acts as an anchor for the search, which will retrieve all Locations in the given context such that the given Relationship holds. The animation procedure uses this information to generate a search key for rules in the Location Relationship ruleform (Figure 8b) that define what conditions must be true to satisfy the user's query. Figure 8b shows the rules that would be selected for a particular genomic Location overlap Relationship. The considerations of these rules, defining the conditions that exist between query Location and retrieved Location, are used to dynamically generate an SQL statement that retrieves the requested Locations. These are then used to query another ruleform to find BioEntities that are located at these Locations; this information is then displayed to the user in a web browser interface (Figure 9).

Nothing in either the structure of the Location Relationship ruleform or the code of the animation procedure is specific to genomic Locations. The end user could add Location Relationship definitions for genomes, freezers, or cells, and those would be immediately queryable within the user interface to the Location procedures. By storing this information as rules rather than in code, the biologist end user has great flexibility in adjusting the system to their needs. In our project, we have most extensively used this capability to correlate results of our proteogenomic mapping with existing annotation sets in our database, to be reported in a separate publication.

### Example 5: Animation procedure for deductive inference

Expressing everything as a rule in the system facilitates automatic deductive inference procedures that can transform implicit assumptions and relationships into explicit rules. For example, "inverse" rules can automatically be generated; if "Serine is-a Polar Amino Acid", then "Polar Amino Acid includes Serine" can be inferred. Rules can also be chained together. A rule may declare that "Gene X encodes-product Protein A", and that "Protein A is-a methyltransferase". If the Relationships "encodes-product" and "is-a" are declared to be validly combinable to deduce the resulting Relationship of "gene-type", chaining these two rules together makes explicit that "Gene X gene-type methyltransferase". Such a deductive process serves first as a mechanism for consistency checking, and secondly to uncover new connections in the data that may have been implicit but not obvious.

**Figure 10:**
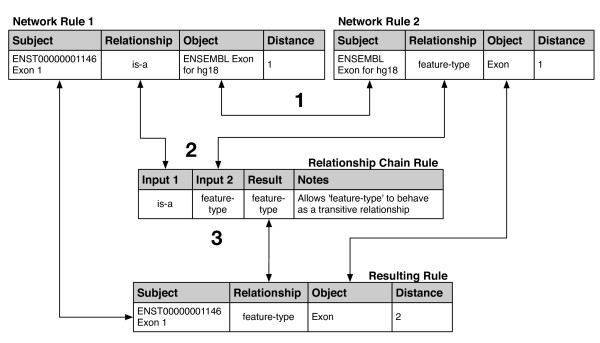
**Network ruleform propagation**. To generate new Network rules, existing rules are combined in logically consistent ways. In Step 1, two BioEntity Network rules are selected to potentially perform a deduction, based on the Object of Rule 1 and the Subject of Rule 2 being the same (BioEntity Network rules are shown, but the principle applies to any Network Ruleform). Deduction can only be carried out if there exists a Relationship Chain rule whose Input 1 and Input 2 fields match the Relationship fields of the two Network Rules, as shown in Step 2. The Relationship Chain ruleform defines rules for composing Relationships together. Here, the selected Relationship Chain Rule expresses the situation that if "A is-a B", and "B has a feature-type of C", then A's feature-type is also deduced to be C. If these conditions are met, then the new rule is generated at Step 3, consisting of the Subject of Rule 1, the Result of the chain rule, and the Object of Rule 2. Note that the Distance field of the resulting rule is the sum of the distances of the two input rules. Users can control how Relationships behave in combination with each other by specifying the appropriate rules in the Relationship Chain ruleform. For example, the "is-a" Relationship can be made transitive by creating a Relationship Chain rule where the Input 1, Input 2, and Result fields are all set to "is-a".

We implemented a general deductive inference animation procedure for each of our network ruleforms. Inverse rule generation is driven by the Relationship of the network rule, which by explicit definition knows its corresponding inverse Relationship (Figure 4b); the procedure simply inserts a new rule using this inverse Relationship and swapping the subject and object of the original rule. It also chains network rules together in logically valid ways to deduce new relationships, governed by the Relationship Chain ruleform. An overview is shown in Figure 10. The animation procedure assembles pairs of network rules based on whether the object of the first rule is equal to the subject of the second rule (Figure 10, Step 1). For each pair, it is then determined whether there is a Relationship Chain rule that pertains to the combination of Relationships represented (Figure 10, Step 2). If yes, a new rule can be deduced by assembling pieces of the input rules and the matching chain rule (Figure 10, Step 3).

The nature of the deduced rule is dependent on the rules inspected in the course of the deduction; the animation procedure simply carries out a series of mechanical database queries and manipulations, purposefully ignorant of the details of what it is computing. Hence, we can easily tell the system that the "is-a" Relationship is transitive by inserting an appropriate Relationship Chain rule whose "Input 1", "Input 2", and "Result" columns are all set to "is-a". Thus, we do not have to code a special case into the deduction procedure to deal with transitive "is-a" relationships, and deduction using other Relationships with more specific meaning (e.g., "part-of", "feature-type", and "file-type") are easily modified by changing the meta-rules.

One practical example of this is that if the system has a rule linking a specific exon to a particular group of exons (e.g., a particular gene), the system can automatically deduce its putative membership in other applicable groups (e.g., homologous genes in other species) using the network structure and the Relationship Chain rules for group membership. By reviewing such deductions, a user may gain new insights into previously opaque relationships. And if a deduced rule is thought contradictory or incorrect vis-a-vis known facts, then this points to the incorrectness of the premises (rules) from which it was derived. Performing that kind of review can be a powerful method for checking the consistency of data and assumptions in the system.

## Discussion

A goal of Ultra-Structure development is to converge upon a deep structure sufficiently general to represent a broad domain of interest, such as systems biology research. The expectation is that the deep structure will be arrived at iteratively, through testing implementations, experimenting, and redesigning. That has been our experience in implementing this system. For example, in an earlier iteration, there was no explicit ruleform for representing locations; we represented locations as BioEntities. Without a separate concept of Location, the system mixed the concept of coordinates of a genomic feature like an exon, with the concept of the exon itself. As we began doing calculations that were unique to location information, such as computing "containment" relationships, it became clear that our original deep structure (set of ruleforms) was too limited. By separating the concept of a thing like an exon into the BioEntities ruleform, and its location into a new Locations ruleform, our system made a lot more sense. At the same time, the development of the Location-related ruleforms opened up a greater set of capabilities for other purposes. This process illustrates Long's hypothesis that notational systems used to represent information can often present inherent limitations of which users are unaware, and that switching the underlying notation often resolves problems and presents new possibilities [22].

Redesigning the ruleforms in Ultra-Structure has impact throughout the system (e.g., on any existing middle structure), and is much like any database redesign, in that it consumes time and effort. The key with Ultra-Structure is then to converge on a foundational deep structure relatively early in the system's evolution. If the result is sufficiently general, further redesigns are minimized, even if very different needs arise. An example is our evolution of the system to represent gene annotations on DNA in two distinct parts: the conceptual entities themselves (genes, exons, introns, etc.) and networks thereof, and the discrete locations of those entities (in arbitrary coordinates). If genome coordinate systems later change, or gene concepts change, this underlying structure is unlikely to need major redesign. For example, a great deal of work is now invested in characterizing methylation patterns on the histones around which DNA is wound, since this appears to have significant regulatory effects on which genes are expressed or when. Hence, there may soon come a time at which annotations will need to include histone methylation patterns. We cannot anticipate the precise way that these will be represented, but it is likely that the system is now general enough to readily accommodate this information without modification to the rule forms.

As the deep structure has emerged and stabilized, subsequent modifications to the system have revolved increasingly around the creation of new rules (middle structure). When we were in the phase of implementing the ruleforms, the Ultra-Structure approach seemed slow and difficult to implement, perhaps due to our lack of experience with it. The high-level perspective required for Ultra-Structure design is quite different from the more standard design methodologies we were accustomed to. Fortunately, as an appropriate and general implementation of ruleforms and animation procedures was developed, we saw ever-increasing efficiency from our efforts, since each implemented part of the deep structure can be utilized for many purposes within the system, amortizing the programming and design workload. An example is the time spent designing and redesigning our representation of Locations. Initially this took longer to get right in Ultra-Structure than it would have using the standard approach of designating specific columns in specific database tables to represent features such as exon start and stop positions. But now that it is in place, nearly any type of Location calculation can be readily implemented. We could use the system's deductive capability to determine anything from which planet a particular sample is located on, to (perhaps more usefully) which organelle a protein is expressed inside of, or which freezer has a patient specimen. Little if any additional programming will be required, excepting the case where we realize our representation is not general enough. In that case, some redesign work may be applied, but with the benefit of further generalizing the system.

An example of this kind of generality is in our recording of sequence tags. Sequence tags are short subsequences of a larger peptide (an amino acid sequence of approximately 5–20 residues), and can be used in conjunction with other information to help identify that larger peptide. In early iterations of our system, we stored sequence tags as textual attribute values on peptide BioEntities, e.g., "DNW" as part of the peptide "DNWDSVTSTF". After creation of the Location ruleforms, we realized that sequence tags could be recast in the same way as any other genome annotation, with a BioEntity representing the existence of a specific tag, and a set of Location entries that describe where the tag is located relative to its parent peptide sequence. We defined new "Peptide Location" rules in the middle structure of the Location Context table, with coordinates for the peptide, start, and stop positions. These rules echo those used to record genomic locations, but without the Strand coordinate (since amino acid sequences are single-stranded). Now, sequence tags are handled like any other annotations on a sequence, except that these are annotations relative to a peptide sequence, whereas genes, exons, and the like are annotations relative to a chromosomal sequence. Interpreting sequence tags as just another type of sequence annotation is the kind of insight that Ultra-Structure was designed to reveal, bringing implementation efficiency and increased semantic clarity.

Making explicit the information about *what *each thing is in the BioEntities and BioEntities Network table, along with *where *each thing is in the Locations-related tables, improves the readability and understandability of the data in the system for human users. This is important because a key objective of Ultra-Structure design is to allow subject experts to directly interact with the rules of a system, without having to explain themselves to a programmer or rely on adequate documentation of existing systems to discover what the rules are. Achieving this makes systems more transparent and eliminates one of the greatest sources of error in systems: the communication between subject experts and computer experts generically referred to as "requirements analysis". This allows subject experts to define their own "terms of art" as needed.

Ultra-Structure provides the ability to readily adapt to evolving requirements and conceptual change in the real world. For example, the classical conception of a gene is that it comprises a contiguous region on one chromosomal strand, yielding a contiguous transcript which then undergoes splicing to produce the mature RNA through the excision of intron regions. This was the basis for our representation of genes in our original database system. Recently, new conceptual challenges to this classical model have arisen, including discovery of significant numbers of gene regions that produce distinct but overlapping transcripts, nested transcripts, and in some cases, mature RNAs resulting from trans-gene splicing [30]. Our original representation of a gene, with a single start and stop site bounding the transcript, would not readily admit oddities like trans-splicing. However, our Ultra-Structure system represents genes as just instances of BioEntities, each of which can be networked to some set of exons (also BioEntities), themselves each of which can have arbitrary location information associated and/or network relationships to other annotations, such as identified MS/MS peptides. This scheme accommodates nearly any possible gene or transcript architecture. The Ultra-Structure system stores trans-spliced transcripts in exactly the same way it stores more canonical, ORF-based transcripts. In fitting with Long's original claims, we have found that our Ultra-Structure system accommodates changing concepts, procedures, and analyses quite well.

The high degree of modifiability of the system has resulted in other benefits. One instance of this was the implementation of computing containment relationships on genomes, such as finding which overlapping gene annotations contain a given exon or peptide location. Initially the rules were set up to analyze containment relationships only on the same strand of a chromosome. At one point we wanted to examine whether there were also any annotations of interest in the same region, but on the opposite strand. We added this capability to the system with just four new middle structure rules added to the database: one to define a Relationship ("contains-(ignoring-strand)") and three to define the conditions for the Relationship (i.e., that the start and stop positions of returned Locations be between the start and stop of the initial query Location, ignoring the strand). This was a simple procedure that could be performed directly and quickly by the end user with no programing or other system alteration.

Performance of our initial prototype system is slower than if traditional ER modeling techniques were used. This is a well-known aspect of systems using decomposed schemas (EAV/CR, RDF triple stores, etc.) in that "entity-centered" queries ("Find all information about Sample 123") perform on par with ER-modeled systems, but "attribute-centered" queries ("Find all lab samples with purity > 98% collected at 37°C") perform more slowly, due to repeated self-joins. Our work has not focused on performance optimizations yet, but this is the focus of ongoing work. Potential solutions exist, ranging from index tuning, alternative query techniques, system configuration tuning, and hardware improvements [31]. Additionally, performance advances in other reasoning systems (particularly Semantic Web-related systems) can potentially help here.

Systems biology is a rapidly changing field, and perhaps one the greatest obstacles to progress is the challenge of effective data management, integration, and analysis across very large, heterogeneous, and complex data sets. Our proteogenomic mapping project presents a microcosm of these challenges with its need to integrate complex genomic and proteomic data sets, all the while keeping up with the rapidly changing technologies in both fields. At present, our prototype Ultra-Structure system is already yielding significant payoff for this task. But perhaps more significantly, it now provides a platform that can be readily adapted to other challenges. That is not just theoretical; we are now beginning to apply the system to other tasks such as managing and analyzing microbiome data and managing and analyzing data from a next-generation sequencing facility. The efforts invested in the deep structure for proteogenomics have immediate application in these other domains.

## Conclusion

Our move to the Ultra-Structure system was not without challenge. Thinking in the "Ultra-Structure way" was initially difficult, and progress was slow. At times we wondered whether a payoff would arrive, or whether we should just go back to the original, more standard approach. But once we reached a threshold of having a stable deep structure, progress has accelerated. Now it is becoming increasingly difficult for us to envision going back to our pre-Ultra-Structure methods, because we see payoffs not only in terms of implementation efficiency, but also in helping us think about our data management and analyses in new ways. This leads us to ponder the question originally raised by Long: could it be that the abstractions and notational systems we have been using were limiting forward progress, without us knowing it? Biological research is no longer content with just studying bits and pieces of an organism or cell, but is instead focused on examining the interactions and dynamics of entire biological systems. Perhaps the needs of the field have outgrown the traditional tools used to represent and analyze biological data. We wonder: will new mental tools like Ultra-Structure design clarify or elucidate aspects of biology that may be concealed by more traditional abstractions? If "complexity" is another way of saying "we don't understand", will new abstractions help overcome barriers to understanding in biology? Ultra-Structure may, at least partially, answer these questions with a "yes". It remains to be seen whether it is the ultimate solution, but so far it has moved us in those directions significantly from where we were before.

## List of Abbreviations Used

EAV: entity-attribute-value; ER: entity-relationship; GFS: Genome Fingerprint Scanning; MS: mass spectrometry; MS/MS: tandem mass spectrometry; ORM: object-relational mapping; RDBMS: relational database management system.

## Authors' contributions

CWM developed the database, web interface, and associated software, populated the system with middle structure rules, and drafted the manuscript. JGL developed Ultra-Structure theory, created an initial system prototype, consulted in the initial stages of the project, and reviewed the manuscript. BMH participated in the conception of the project, provided feedback during the research, and reviewed the manuscript. MCG conceived of the project, provided advice and guidance on the system architecture, and assisted in the drafting and revision of the manuscript. All authors have read and approved the final manuscript.

## Supplementary Material

Additional file 1 **Example SQL Queries**. Contains some sample SQL queries that the Ultra-Structure system uses. These queries would not be used directly by end-users; instead, they are used or generated by animation procedures. Click here for file

Additional file 2 **Ultra-Structure Prototype**. Distribution of prototype code. Instructions for use are contained inside. Click here for file
