# Supplementary material for: Ultra-Structure database design methodology for managing systems biology data and analyses
Source: BMC Bioinformatics. 2009 Aug 19;10:254. doi: 10.1186/1471-2105-10-254 (PMC2748085; doi:10.1186/1471-2105-10-254)
Supplement: Additional file 1 — Example SQL Queries. Contains some sample SQL queries that the Ultra-Structure system uses. These queries would not be used directly by end-users; instead, they are used or generated by animation procedures. [file 1471-2105-10-254-S1.pdf]

Example SQL Queries

Though users will not be expected to interact with the system directly via SQL, some example queries are provided here to offer insight as to the inner workings of our Ultra-Structure system. SQL like that shown here is used or generated by the animation software.

Find the BioEntity named "Carbon":

```
SELECT * FROM bioentity WHERE name='Carbon';
```

Find all BioEntities with a mass of 1234.25:

```
SELECT b.*
FROM bioentity AS b
JOIN bioentity_attribute AS ba ON b.id = ba.bioentity
JOIN attribute AS a ON a.id = ba.attribute
WHERE ba.numeric_value = 1234.25
  AND a.name = 'Molecular Weight'
;
```

Find the Location(s) on the forward strand of chromosome 20 that lie between position 57,156,249 and at 57,156,276

Note: Text values shown below (e.g., 'Base Sequence','Strand', etc.) are for clarity and conciseness only; in the actual system, numeric identifiers are used.

```
SELECT location.*
FROM location
     JOIN location_attribute AS la1 ON location.id =
la1.location AND la1.attribute = 'Base Sequence'
     JOIN location_attribute AS la2 ON location.id =
la2.location AND la2.attribute = 'Strand'
     JOIN location_attribute AS la3 ON location.id =
la3.location AND la3.attribute = 'Start'
     JOIN location_attribute AS la4 ON location.id =
la4.location AND la4.attribute = 'Stop'
WHERE la1.bioentity_value = 'Chromosome 20'
  AND la2.bioentity_value = 'Forward Strand'
  AND la3.integer_value >= 57156249
  AND la4.integer_value <= 57156276
  AND location.context = 'Human Genome Draft 18 Coordinate'
;
```
